# Evaluating the Endocytosis and Lineage-Specification Properties of Mesenchymal Stem Cell Derived Extracellular Vesicles for Targeted Therapeutic Applications

**DOI:** 10.3389/fphar.2020.00163

**Published:** 2020-03-03

**Authors:** Chun-Chieh Huang, Miya Kang, Raghuvaran Narayanan, Luisa A. DiPietro, Lyndon F. Cooper, Praveen Gajendrareddy, Sriram Ravindran

**Affiliations:** ^1^Department of Oral Biology, College of Dentistry, University of Illinois at Chicago, Chicago, IL, United States; ^2^Department of Periodontics, College of Dentistry, University of Illinois at Chicago, Chicago, IL, United States

**Keywords:** exosome, extracellular vesicles, mesenchymal stem cells, differentiation, endocytosis

## Abstract

Mesenchymal stem cells (MSCs) are multipotent cells with regenerative and immunomodulatory properties. Several aspects of MSC function have been attributed to the paracrine effects of MSC derived extracellular vesicles (EVs). Although MSC EVs show great promise for regenerative medicine applications, insights into their uptake mechanisms by different target cells and the ability to control MSC EV properties for defined function *in vivo* have remained elusive knowledge gaps. The primary goal of this study is to elucidate how the basic properties of MSC derived EVs can be exploited for function-specific activity in regenerative medicine. Our first important observation is that, MSC EVs possess a common mechanism of endocytosis across multiple cell types. Second, altering the MSC state by inducing differentiation into multiple lineages did not affect the exosomal properties or endocytosis but triggered the expression of lineage-specific genes and proteins *in vitro* and *in vivo* respectively. Overall, the results presented in this study show a common mechanism of endocytosis for MSC EVs across different cell types and the feasibility to generate functionally enhanced EVs by modifications to parental MSCs.

## Introduction

Human mesenchymal stem cells (HMSCs) are multipotent somatic stem cells that can be isolated from a variety of tissues such as the bone marrow, adipose tissue, and dental pulp. The regenerative, protective, and anti-inflammatory properties of HMSCs especially bone marrow derived HMSCs are well documented (Yu et al., 2014; Kim and Park, 2017) and make HMSCs attractive cells for regenerative therapies. As of 2016, about 493 clinical trials that used HMSCs were reported in the National Institutes of Health (NIH) clinical trials database (Squillaro et al., 2016). However, issues such as donor dependent variability, cellular viability, poor attachment, and aberrant differentiation have posed significant hurdles for the use of HMSCs in clinical treatment (Zhang et al., 2015; Kim and Park, 2017).

Many existing tissue-engineering approaches focus on delivery of selected proteins (growth factors, transcription factors etc.) or nucleic acids to host or implanted stem cells to achieve lineage specific differentiation. A variety of techniques ranging from exogenous addition of growth factors and controlled release devices [reviewed in (Mao and Mooney, 2015)] to utilization of engineered biological and synthetic nano-vesicles such as liposomes and polymeric vesicles (Li et al., 2012; Soltani et al., 2015) have been investigated to deliver morphogens. Although the single morphogen system shows initial promise, when applied clinically, issues such as dosage, specificity, ectopic effects, toxicity, and immunological complications have posed significant restrictions to clinical efficiency as well as translational potential (Soltani et al., 2015). Therefore, a sophisticated system that is biomimetic in nature, provides necessary cues in physiologically relevant amounts and avoids the limitations of the single morphogen system is required. Extracellular vesicles (EVs)/exosomes can satisfy these criteria (Marcus and Leonard, 2013).

EVs are nano-vesicles (40–150 nm) secreted by cells to facilitate intracellular communication (Park et al., 2008). As these vesicles pinch off or fuse with the plasma membrane of the cell prior to release, their lipid bilayer membrane contains components of the parental cell’s plasma membrane (Stoorvogel et al., 2002). Within the EV, RNA [both messenger RNA (mRNA) and microRNA (miRNA)], cytosolic proteins, as well as transmembrane proteins are present (Thery et al., 2002). These nano-packets of information are endocytosed by effector cells to trigger a cellular response designated by the parental cell to the target cell (Valadi et al., 2007; Svensson et al., 2013). Although originally believed to be mediators of cellular homeostasis by secreting cellular waste (Johnstone et al., 1991), the past decade study of EVs demonstrate their specific roles in modulating cellular function in immunology, cancer biology, and regenerative medicine (Johnstone et al., 1991; Azmi et al., 2013).

Recent evidence suggests that several of the beneficial effects of human mesenchymal stem cell (HMSC) therapy can be attributed to paracrine effects of the HMSC secretome (Dai et al., 2007; Gnecchi et al., 2008; Yao et al., 2015). More specifically, HMSC derived EVs have been implicated as the principal active agents of the HMSC secretome (Lai et al., 2010; Mokarizadeh et al., 2012; Reis et al., 2012). A recent study highlighted that HMSC derived exosomes possess better anti-inflammatory properties compared to HMSC derived microparticles (Cosenza et al., 2018). Our recent studies have shown that bone marrow and dental pulp HMSC derived EVs can be used to induce osteogenic and odontogenic differentiation of naïve HMSCs respectively (Huang et al., 2016; Narayanan et al., 2016). Additionally, a recent study by Narayanan et al. indicates that HMSC EV function supersedes the extracellular matrix (ECM) derived signals indicating the potent nature of EV signaling (Narayanan et al., 2018). These and many other studies implicate HMSC derived EVs as effective tools in clinical efforts to control inflammation and regenerative therapy and in the treatment of disease.

The paracrine aspect of HMSC function involves the directed uptake of HMSC derived EVs by target cells. Further, the multilineage differentiation potential of HMSCs suggests that lineage specific function could be reflected as lineage specific exosomal effects on naïve target cells. Harnessing the fundamental mechanistic features of EV-mediated signaling can be turned into an application-specific tool to direct lineage specific tissue repair/regeneration and disease treatment. With these goals in mind, the present study characterizes basic mechanistic aspects of HMSC EV function.

## Materials and Methods

### Cell Culture

Human bone marrow derived primary HMSCs (HMSCs) were purchased from ATCC and Lonza. Over the course of this study, HMSCs from at least three individual lots spanning at least three donors were utilized. These cells were cultured in αMEM (Gibco) containing 20% fetal bovine serum (FBS, Gibco), 1% L-Glutamine (Gibco), and 1% antibiotic-antimycotic solution (Gibco). For induction of differentiation of HMSCs into osteogenic (Ravindran et al., 2012), chondrogenic (Ravindran et al., 2015) and adipogenic (Scott et al., 2011) lineages, the growth medium was supplemented with growth factors and differentiating agents as per the indicated published and standardized protocols. Briefly, osteogenic differentiation was induced by culturing the cells in αMEM growth medium containing 100 µg/ml ascorbic acid (Sigma), 10 mM β-glycerophosphate (Sigma), and 10 mM dexamethasone (Sigma) for 4 weeks. Chondrogenic differentiation was induced by culturing the cells in αMEM basal medium containing 1 µM dexamethasone, 50 µg/ml ascorbate-2-phosphate (Sigma), 1% ITS premix (BD Biosciences), 1% FBS, and 10 ng/ml TGFβ1 growth factor (Sigma) for 4 weeks. Adipogenic differentiation was induced by culturing the cells in growth medium containing 10 μg/ml insulin (Sigma), 500 µM isobutyl-l-methylxanthine (Sigma), 100 μM indomethacin (Sigma), and 1 μM dexamethasone for 4 weeks. Human gingival keratinocyte cell line TIGK was cultured as per standardized protocols using the basal medium LM-0004 (Lifeline Cell Tech) supplemented with 10% FBS and LS-1030 cell supplement kit (Lifeline Cell Tech). J774A.1 mouse monocyte-like cells were cultured in DMEM (Gibco) basal medium supplemented with 10% FBS and 1% antibiotic-antimycotic solution.

### Extracellular Vesicle Isolation and Characterization

EVs were isolated from the culture medium as per our previously published and standardized protocols (Huang et al., 2016; Narayanan et al., 2016). Briefly, HMSCs were washed in serum free medium and cultured under serum free condition for 24 h. If they were subjected to supplementation for altering cell state, the supplementation was maintained with only FBS being removed. The culture medium was harvested, removed of cell debris by centrifugation (1,500xg) and EVs were isolated using the ExoQuick TC isolation reagent (System Biosciences) as per the manufacturer’s recommended protocols. To maintain consistency, the isolated EVs were resuspended in phosphate-buffered saline (PBS) such that each 100 µl of EV suspension contained EVs from approximately 1x10^6^ HMSCs. This equated to a stock concentration of 10,000 particles/µl as determined by nanoparticle tracking analysis (NTA).

The isolated EVs were characterized for number and size distribution and presence of membrane markers by NTA, immunoblotting, and transmission electron microscopy (TEM) as per established standards (Thery et al., 2018). For NTA, a 1/100 dilution of the EV suspension was analyzed in the Nanosight NS-300 instrument to obtain the size distribution plot. For quantitative experiments, the EV concentration (particles/ml) was also measured by NTA and equal number of EVs were used for each experiment.

For immunoblotting, exosomal proteins were isolated in radioimmunoprecipitation assay (RIPA) buffer and 10–20 µg of EV protein isolate was resolved by sodium dodecyl sulfate polyacrylamide gel electrophoresis (SDS-PAGE), transferred onto nitrocellulose membranes and probed with primary rabbit anti-CD63 (1/500, Abcam) and mouse anti-CD9 (1/500, Abcam), mouse anti-BMP2 (1/500, Abcam) antibodies and near infrared dye conjugated secondary antibodies (1/10,000 Licor) as per previously published protocols (Huang et al., 2016; Narayanan et al., 2016). The blots were then dried and imaged using a Licor Odyssey imager. For immunoblotting of the conditioned medium, the medium from which EVs were isolated was dialyzed against deionized water, lyophilized and reconstituted in 1x Laemmli buffer. SDS PAGE and immunoblotting was performed as described previously.

For transmission electron microscopy (TEM), 10 µl of 1/10 dilutions of the EV suspensions were placed on to carbon fomvar coated nickel TEM grids and incubated for 1 h followed by fixing with 4% formalin, washing with double deionized water and air drying. For immunogold labeling of CD63, the EV containing grids were blocked in PBS with 5% BSA, incubated with CD63 antibody (1/100, Abcam) followed by washing and incubation with 10 nm gold tagged secondary antibody (1/1,000, Abcam). The grids were then washed and air-dried. All the grids were imaged using a Joel JEM3010 TEM.

For all the experiments described in this study, EVs isolated from one lot of HMSCs were used for functional experiments on the same lot of HMSCs.

### Quantitative and Qualitative Endocytosis of Human Mesenchymal Stem Cell Extracellular Vesicles

For endocytosis experiments, HMSC EVs were fluorescently labeled using the ExoGlow green labeling kit (System Biosciences) that labels the exosomal proteins fluorescently as per our previously published and standardized protocols (Huang et al., 2016; Narayanan et al., 2016). The EVs were resuspended in PBS at the same concentration as described previously (100 µl corresponding to EVs from 1 million HMSCs).

For quantitative experiments HMSC cells were plated on to 96 well tissue culture plates at a concentration of 10,000 cells per well and incubated for 18 h to facilitate cell attachment. The cells were then incubated with increasing amounts of fluorescently labeled HMSC EVs for 2 h at 37°C. The cells were washed with PBS and fixed in neutral buffered 4% paraformaldehyde. The fluorescence from the endocytosed EVs was measured using a BioTek Synergy2 96 well plate reader equipped with the appropriate filter sets to measure green fluorescence. The results were plotted as mean (+/− SD) normalized fluorescence intensities (normalized to background and no EV fluorescence) as a function of dosage (n = 6 per group).

For quantitative endocytosis blocking experiments, the cells were plated in 96 well plates as described previously or in 12 well culture plates (50,000 cells/well) and prior to EV treatment, were pre-treated with the blocking agents for 1 h as per our previously published protocols (Huang et al., 2016). Cell surface integrins were blocked with 2 mM arginylglycylaspartic acid (RGD) polypeptide (Sigma). Membrane cholesterol was depleted using methyl β cyclodextrin [methyl-β-cyclodextrin (MBCD), Sigma] in a dose dependent manner (0–10 mM). In addition to this, the labeled EVs were pretreated for 1 h with indicated concentrations of heparin (0–10 µg/ml, Sigma) to block the heparin sulfate proteoglycan binding sites on the exosomal membrane. For the qualitative and quantitative experiments, the fluorescently labelled exosomal volume was maintained at 2x saturation volume (determined from the saturation curve. The stock concentration of EV was 10,000 particles/µl) to ensure that saturable levels of HMSC EVs are used in the assay. Treatment with the EV suspension was carried out as described previously and the fluorescence measurement and quantitation and statistical analysis was performed as per published (Huang et al., 2016) and previously described protocols.

For qualitative endocytosis experiments, 50,000 cells (HMSCs) were plated on coverslips placed in 12 well tissue culture dishes. Fluorescently labeled EVs at 2x saturation volume were then added with/without inhibitors as described above and incubated for 2 h in the presence/absence of blocking agents as described above. The cells were then washed, fixed in 4% neutral buffered paraformaldehyde, permeablized, and counter stained using mouse monoclonal anti-tubulin antibody (1/2,000, Sigma), rabbit polyclonal anti-caveolin1 antibody (1/100, Santa Cruz Biotechnology), or rabbit polyclonal anti-clathrin antibody (1/100, Santa Cruz Biotechnology) followed by treatment with TRITC labeled anti mouse/rabbit secondary antibody. The coverslips were then mounted using mounting medium containing 4′,6-diamidino-2-phenylindole (DAPI) (Vector Labs) to label the nuclei and imaged using a Zeiss LSM 710 Meta confocal microscope.

### Extracellular Vesicle Mediated Human Mesenchymal Stem Cell Differentiation

HMSCs were differentiated as described under the cell culture methods section and EVs from the differentiated HMSCs were isolated as described under the isolation section. The isolated EVs were characterized for size and the presence of exosomal markers as described under the characterization section. For *in vitro* differentiation experiments, naïve HMSCs (250,000 cells per 1 cm x 1 cm collagen sponge) were embedded in type I collagen sponges in quadruplicates. Clinical grade collagen sponges (Zimmer collagen tape) were used for these experiments. 2x saturation volume of the different EVs (osteogenic, chondrogenic, and adipogenic) were then added to the cells and incubated for 72 h. The saturation volume was determined by the quantitative dose dependence endocytosis experiment described in the previous section. The saturation was reached at 20 µl of standardized EV suspension per 10,000 HMSCs. NTA was used to measure the amount of EVs and this amounted an average of 10,000 EV particles/µl of standardized EV suspension from HMSCs. 1x10^8^ EV particles was used per group in this experiment. Untreated cells received PBS treatment of equal volume. Post-72 h, RNA was isolated from the embedded HMSCs followed by cDNA synthesis and qPCR for selected marker genes for osteogenic, chondrogenic, and adipogenic differentiation as per our previously published protocols and primer sequences (Huang et al., 2016; Narayanan et al., 2016).

### Mouse Subcutaneous Implantation Experiments

All *in vivo* experimentation was performed in either immunocompromised mice (1-month old mice, Charles River Labs) or Sprague Dawley rats (250–300 g, Charles River Labs) as per protocols and procedures approved by the University of Illinois animal care committee (ACC). All animals were housed in appropriate cages in temperature and humidity-controlled facilities. Food and water were made available *ad libitum*.

The ability of EVs from differentiated HMSCs to induce lineage specific differentiation of naïve HMSCs was evaluated *in vivo* in an immunocompromised mouse subcutaneous implantation model. Briefly, 1x10^6^ HMSCs were seeded on to a 1 cm x 1 cm square of clinical grade collagen tape (Zimmer) with 2x saturation volume (approximately 4x10^8^ EVs) of respective control (naïve HMSC EV) or experimental EV (osteogenic, chondrogenic, or adipogenic) suspension and implanted within the subcutaneous pocket bilaterally on the back of immunocompromised mice. The mice were anesthetized by intraperitoneal injection of ketamine (80–100 mg/kg)/xylazine (10 mg/kg). A 1.5 cm incision was made on the back along the midline and the control or experimental scaffolds were placed bilaterally within the subcutaneous pocket. All experiments were performed in quadruplicate. Four weeks post-implantation, the animals were sacrificed by carbon dioxide asphyxiation followed by cervical dislocation. The scaffolds were extracted, fixed in neutral buffered 4% paraformaldehyde, embedded in paraffin, and sectioned in to 5 µm sections. The sections were then immunostained fluorescently for marker proteins as per previously published protocols (Huang et al., 2016; Narayanan et al., 2016), mounted, and imaged using a Zeiss LSM 710 laser scanning confocal microscope. All primary antibodies were purchased from Abcam and were used at a dilution of 1/100 of the stock solution. The secondary anti-mouse fluorescein isothiocyanate (FITC) and anti-Rabbit TRITC were obtained from Sigma and were used at a dilution of 1/200.

### Statistical Analysis

For all experiments the normal distribution of the data was evaluated using the Shapiro-Wilk test. Following confirmation to normal distribution, appropriate parametric tests were used to calculate statistical significance. For experiments involving comparison of just two groups, student’s t-test with a confidence interval of 95% was utilized. For the experiments involving comparison of more than two groups, one-way ANOVA was performed with a confidence interval of 95%. Following this, pairwise comparisons were performed using Tukey’s *ad-hoc* method with a confidence interval of 95%. Statistical analyses were performed using either SPSS software or Microsoft Excel.

## Results

### Characterization of Extracellular Vesicles

**Figure 1:**
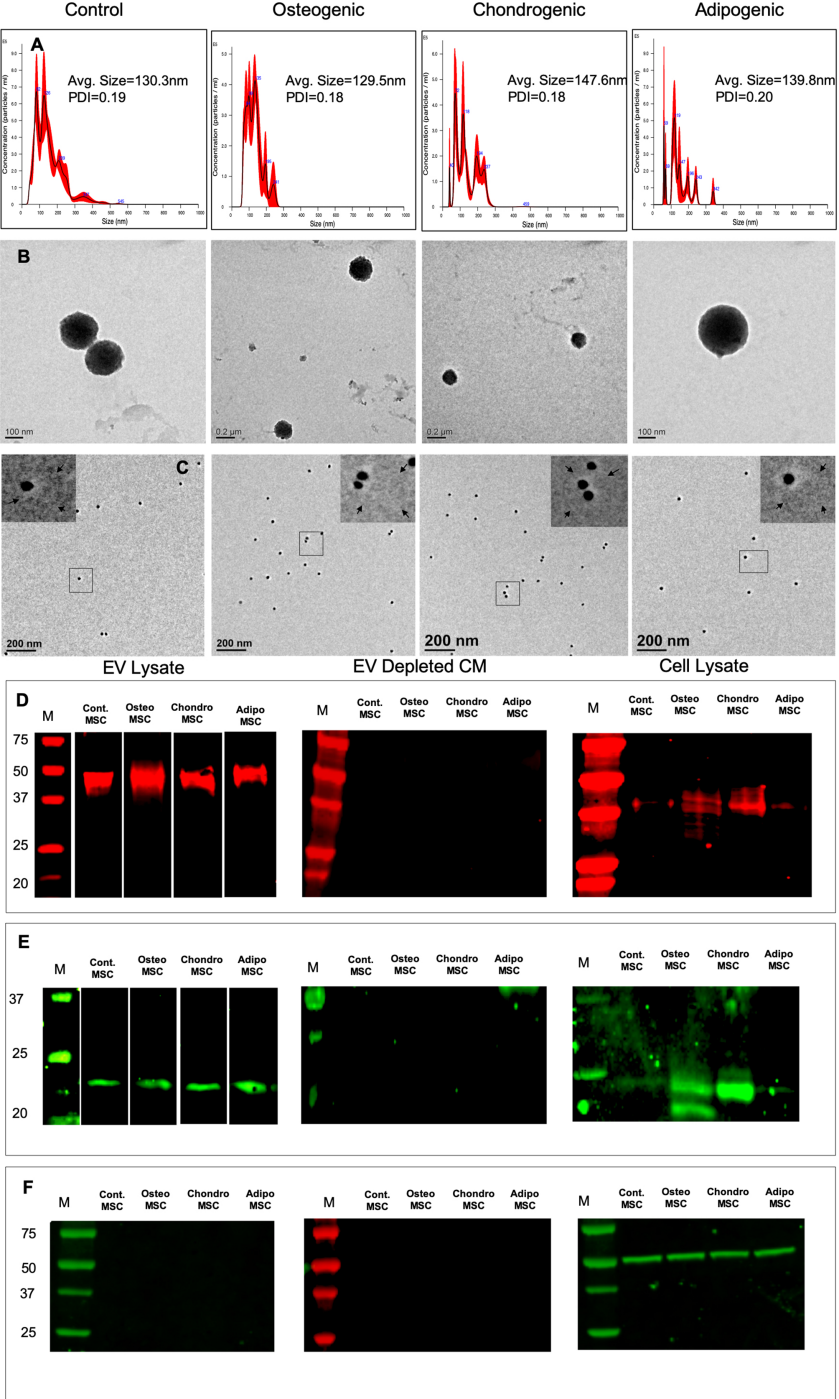
Isolation and characterization of extracellular vesicles (EVs): **(A)** Representative nanoparticle tracking analysis (NTA) plots of EVs isolated from naïve, osteogenic, chondrogenic, and adipogenic human mesenchymal stem cells (HMSCs). **(B)** Representative transmission electron microscopy images of the EVs isolated from naïve, osteogenic, chondrogenic, and adipogenic HMSCs. **(C)** Representative TEM images of Immunogold labeled (CD63, 20 nm gold particles) EVs from naïve, osteogenic, chondrogenic, and adipogenic HMSCs. The inserts in each of the images represent the boxed area. The arrows in the inserts point to EV membranes. **(D)** Immunoblot of EVs lysates, EV depleted conditioned medium, and cell lysates from naïve, osteogenic, chondrogenic, and adipogenic HMSCs for the presence of CD63 exosomal marker protein. **(E)** Immunoblot of EVs lysates, EV depleted conditioned medium, and cell lysates from naïve, osteogenic, chondrogenic, and adipogenic HMSCs for the presence of CD9 exosomal marker protein. **(F)** Immunoblot of EVs lysates, EV depleted conditioned medium, and cell lysates from naïve, osteogenic, chondrogenic, and adipogenic HMSCs for the presence tubulin.

EVs isolated from HMSCs were characterized for size, shape, and presence of exosomal marker proteins. The isolation procedure did not induce cell death in the source HMSCs (**Supplementary Figure 1**). NTA analysis indicated that the isolated vesicles show a particle size distribution consistent for exosomes (Shah et al., 2018; Thery et al., 2018) (**Figure 1A**). We determined that on average, after our standardized EV dilution (100 µl suspension containing EVs from 1x10^6^ cells), the EV concentration for HMSCs was approximately 1x10^8^ particles/ml of the EV suspension. Osteogenic, chondrogenic, and adipogenic differentiation of HMSCs yielded EVs with a similar average size, but an altered distribution of EV sizes (**Figure 1A**). However, the polydispersity index (PDI) was similar between the different groups (**Figure 1A**). TEM analysis revealed spherical vesicles between 100 and 150 nm in size and positive for CD63 marker (**Figures 1B, C**). Immunoblot analysis indicated the presence of exosomal marker proteins CD63 (**Figure 1D**) and CD9 (**Figure 1E**) in both naïve and differentiated HMSC EVs, but not in the EV depleted conditioned medium. Immunoblotting for tubulin revealed tubulin presence in the cell lysate, but not in the EV lysate and EV depleted conditioned medium (**Figure 1F**). The data presented here indicate that the EVs may be primarily composed of exosomes. However, as the exosomes and other EVs have overlapping properties and as we cannot conclusively determine that the purified vesicles are only exosomes, we will refer to them as EVs throughout this article.

### Endocytosis of Human Mesenchymal Stem Cell Derived Extracellular Vesicles

**Figure 2:**
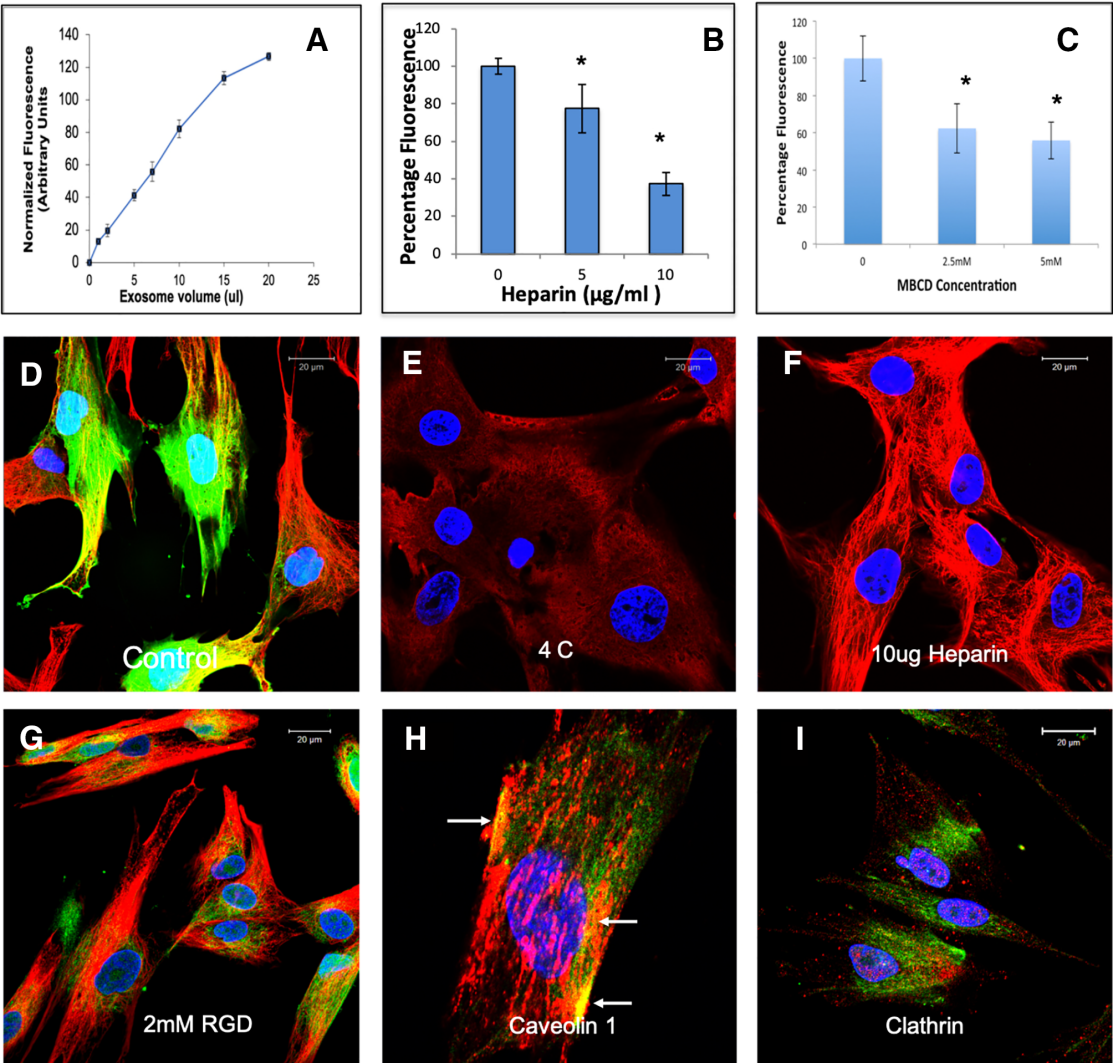
Endocytosis of human mesenchymal stem cell (HMSC) extracellular vesicles (EVs) by HMSCs: **(A)** Graphical representation of dose-dependent and saturable endocytosis of fluorescently labeled HMSC EVs by naïve HMSCs. Data points represent mean fluorescence +/− SD (n=6). The EV volume/particle number was standardized as described under the methods section. **(B)** Graph showing the dose dependent inhibition of HMSC EV endocytosis after pre-treatment of the EVs with heparin to block interaction with the cell surface HSPGs. Data represent mean percentage fluorescence with respect to control +/− SD. **(C)** Graph showing the reduction in HMSC endocytosis after disruption of target cell membrane cholesterol with varying doses of methyl-β-cyclodextrin (MBCD). Data is presented as mean percentage fluorescence with respect to control +/− SD. Representative confocal micrograph depicting the endocytosed fluorescently labeled HMSC EVs within target HMSCs after 1 h of incubation at 37°C. **(E)** Representative confocal micrograph indicating the abrogation of HMSC EV endocytosis when the experiment is performed at 4°C. **(F)** Representative confocal micrograph showing that pre-treatment of EVs with heparin blocks HMSC EV endocytosis. **(G)** Representative confocal micrograph of HMSC EV endocytosis after pre-treatment of the cells with 2 mM arginylglycylaspartic acid (RGD) peptide to block cell surface integrins. In images **(D**–**G)**, green fluorescence represents endocytosed EVs. Red fluorescence represents tubulin counter stain and blue fluorescence indicates 4′,6-diamidino-2-phenylindole (DAPI) nuclear stain. **(H)** Confocal micrograph showing colocalization of endocytosed HMSC EVs (green) with caveolin1 (red). **(I)** Confocal micrograph showing the absence of co-localization between endocytosed EVs (green) and clathrin (red). * represents statistical significance (P < 0.05, ANOVA followed by Tukey’s *ad-hoc*) with respect to control.

EVs from different cell types have been shown to be endocytosed by a variety of mechanisms (Mulcahy et al., 2014). Here, we evaluated the endocytic mechanism of HMSC EVs by target HMSCs. Quantitative endocytosis experiments indicated that HMSC EV endocytosis by HMSCs was a dose dependent and saturable process (**Figure 2A**). When the quantitative and qualitative endocytosis experiments were performed at 4°C using saturable amounts of EVs, EV endocytosis was blocked indicating the temperature and thereby, the energy dependency of the process (**Figures 2D, E**). In an attempt to identify the mode of endocytosis, we evaluated the role of integrins. Published studies have shown that EV endocytosis by dendritic cells is mediated by integrins (Morelli et al., 2004; Mulcahy et al., 2014). Pre-treatment of the target cells with 2 mM RGD peptide to block the cell surface integrins did not block EV endocytosis (comparing **Figures 2D, G**) indicating that integrins are not primary receptors involved in HMSC EV endocytosis.

A recently published study shows that heparan sulfate proteoglycans (HSPGs) are involved in the endocytosis of glioblastoma cell derived exosomes (Christianson et al., 2013). HSPGs act as both receptors and co-receptors on the plasma membrane and are actively involved in endocytosis of several viruses (Shukla et al., 1999; Belting, 2003). Sulfated heparin mimics the extracellular heparan sulfate domains of the HSPGs and can competitively block endocytosis *via* HSPGs by actively binding to the EVs (Christianson et al., 2013). We therefore investigated if HSPGs are involved in the endocytosis of EVs. Pretreatment of the EVs with heparin significantly reduced the endocytosis in both quantitative (**Figure 2B**) and qualitative (**Figure 2F**) experiments suggesting the involvement of membrane heparin sulfate proteoglycan receptors (HSPGs) in the process of EV endocytosis (Christianson et al., 2013).

Depending on the target cell type, exosomes can be endocytosed by either clathrin or caveolin mediated endocytosis (Mulcahy et al., 2014). We performed immunolocalization experiments to evaluate the involvement of a clathrin or caveolin mediated pathway. In these experiments, the endocytosed EVs colocalized with caveolin 1 (**Figure 2H**). Blocking the lipid raft/caveolar endocytosis with MBCD inhibited EV endocytosis significantly (**Figure 2C**). On the other hand, no colocalization with clathrin and the endocytosed EVs was observed (**Figure 2I**).

**Figure 3:**
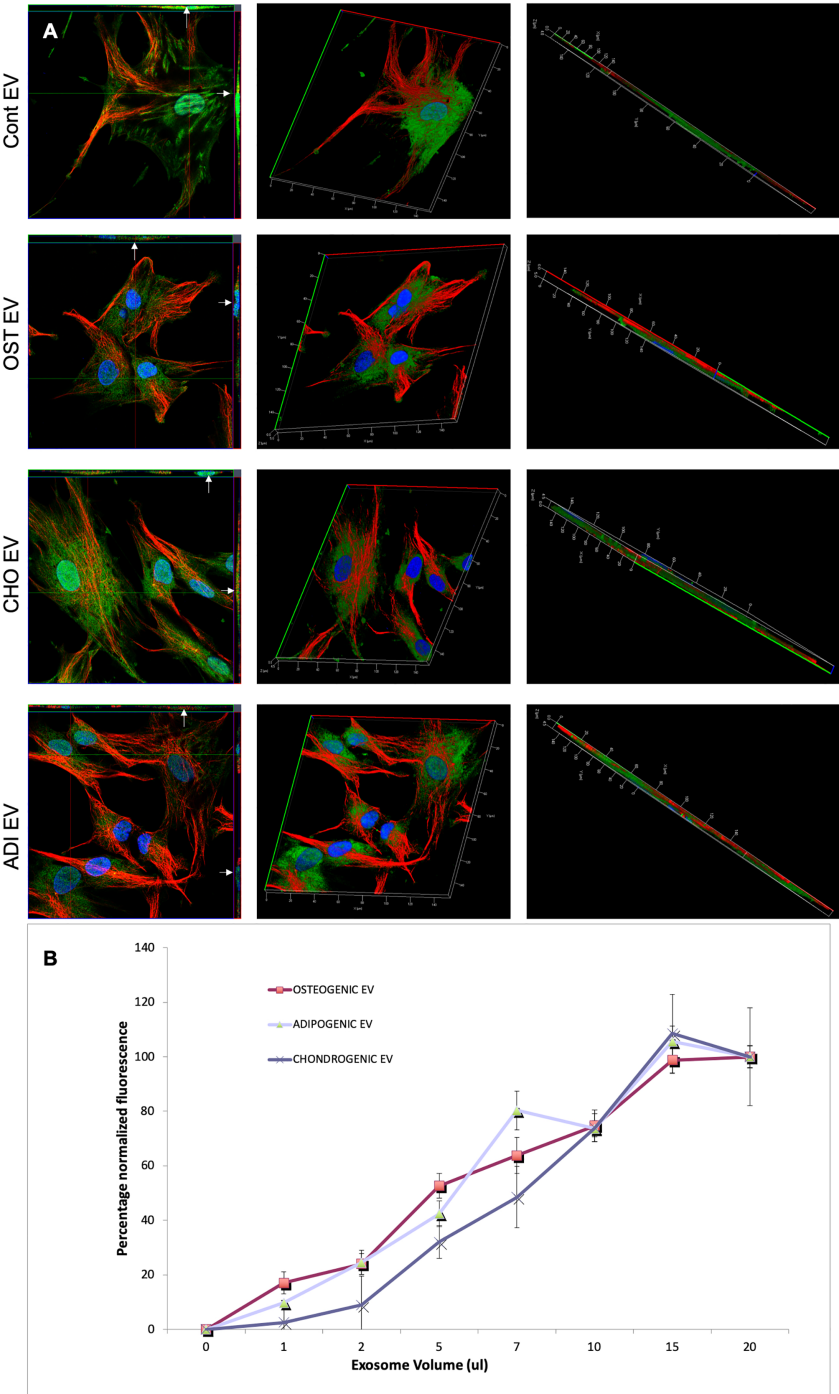
Endocytosis of extracellular vesicles (EVs) isolated from differentiated human mesenchymal stem cells (HMSCs): **(A)** Representative 3D confocal micrographs of fluorescently labeled EVs isolated from control (naïve), osteogenic, adipogenic, and chondrogenic HMSCs endocytosed by naïve HMSCs. The first image in each row is an orthogonal representation of the z-stacks. The arrows point to localization of the EVs within the cells in the x-z and y-z planes. The other images in the rows represent 3D reconstructions of the z-stacks showing the endocytosed EVs within the cells. In all images, green fluorescence represents labeled EVs, blue represents DAPI nuclear stain and red represents tubulin. **(B)** Graph showing dose dependent and saturable endocytosis of EVs isolated from osteogenic, chondrogenic, and adipogenic HMSCs by naïve HMSCs. Data points represent mean percentage fluorescence with respect to the highest concentration +/− SD (n = 6). Note the absence of any significant difference in endocytosis between EVs isolated from the three lineages.

Following these observations on HMSC EV endocytosis, we next evaluated if a change in cell state would affect the endocytosis of lineage specified, HMSC derived EVs. HMSCs were first differentiated along the osteogenic, chondrogenic, and adipogenic lineages. EVs isolated from these cells were harvested and evaluated for dose dependent and saturable endocytosis. **Figure 3A** shows representative confocal images of the endocytosis of different fluorescently labeled EVs by naïve HMSCs. Quantitative endocytosis assays with the different MSC EVs revealed no differences in the dose-dependence or saturation of endocytosis (**Figure 3B**).

**Figure 4:**
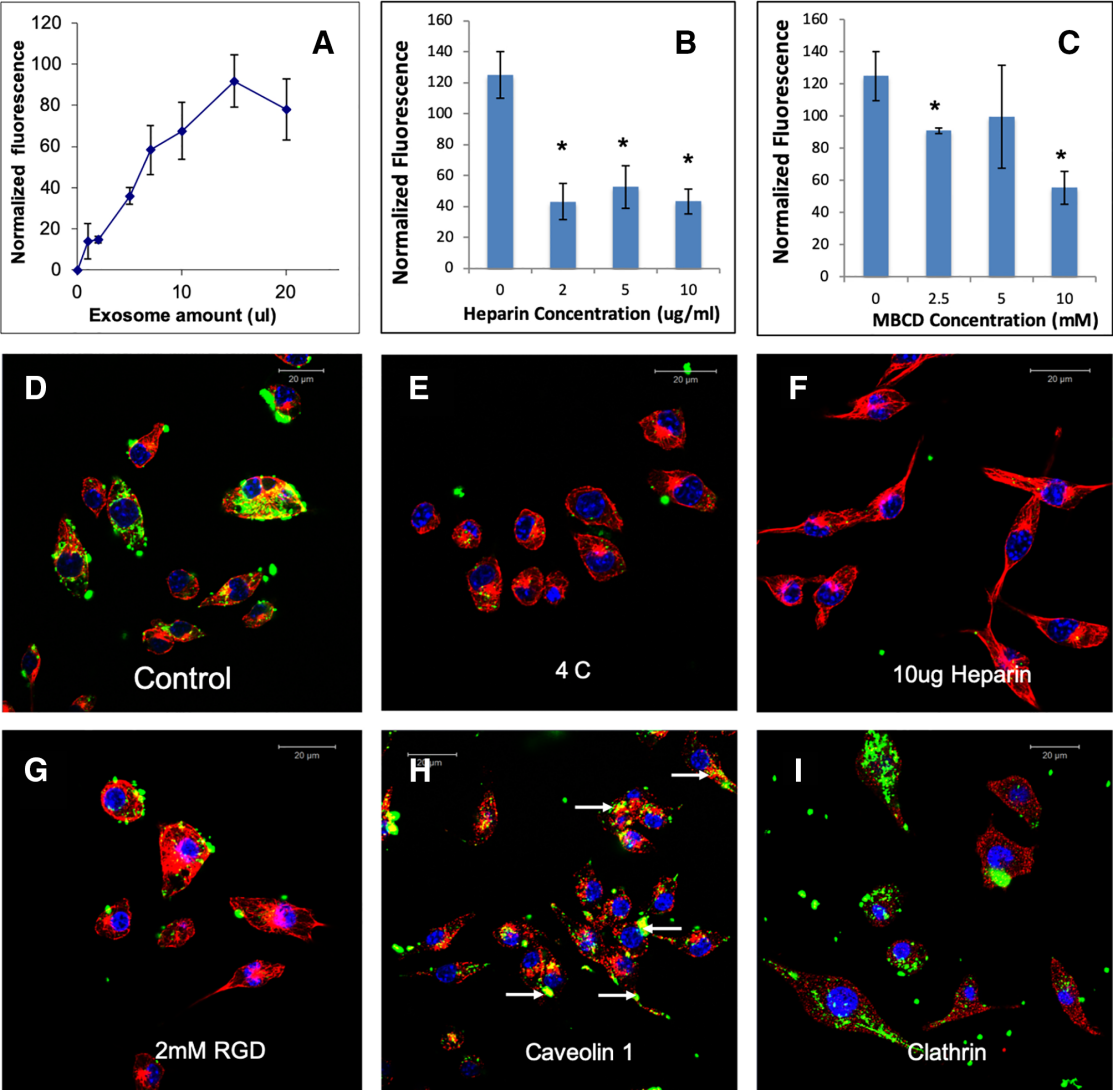
Endocytosis of HMSC extracellular vesicles (EVs) by J774A.1 monocytes: **(A)** Graphical representation of dose-dependent and saturable endocytosis of fluorescently labeled human mesenchymal stem cell (HMSC) EV by J774A.1 cells. Data points represent mean fluorescence (n=6) +/− SD. The EV volume was standardized as described under the methods section. **(B)** Graph showing the dose dependent inhibition of HMSC EV endocytosis after pre-treatment of the EVs with heparin to block interaction with the cell surface heparan sulfate proteoglycans (HSPGs). Data represent mean percentage fluorescence with respect to control +/− SD. **(C)** Graph showing the reduction in HMSC endocytosis after disruption of target cell membrane cholesterol with varying doses of methyl-β-cyclodextrin (MBCD). Data is presented as mean percentage fluorescence with respect to control +/− SD. Representative confocal micrograph depicting the endocytosed fluorescently labeled HMSC EVs within target HMSCs after 1 h of incubation at 37°C. **(E)** Representative confocal micrograph indicating the abrogation of HMSC EV endocytosis when the experiment is performed at 4°C. **(F)** Representative confocal micrograph showing that pre-treatment of EVs with heparin blocks HMSC EV endocytosis. **(G)** Representative confocal micrograph of HMSC EV endocytosis after pre-treatment of the cells with 2 mM RGD peptide to block cell surface integrins. In images **(D**–**G)**, green fluorescence represents endocytosed EVs. Red fluorescence represents tubulin counter stain and blue fluorescence indicates 4′,6-diamidino-2-phenylindole (DAPI) nuclear stain. **(H)** Confocal micrograph showing colocalization of endocytosed HMSC EVs (green) with caveolin1 (red). **(I)** Confocal micrograph showing the absence of co-localization between endocytosed EVs (green) and clathrin (red). * represents statistical significance (P < 0.05, ANOVA followed by Tukey’s *ad-hoc*) with respect to control.

**Figure 5:**
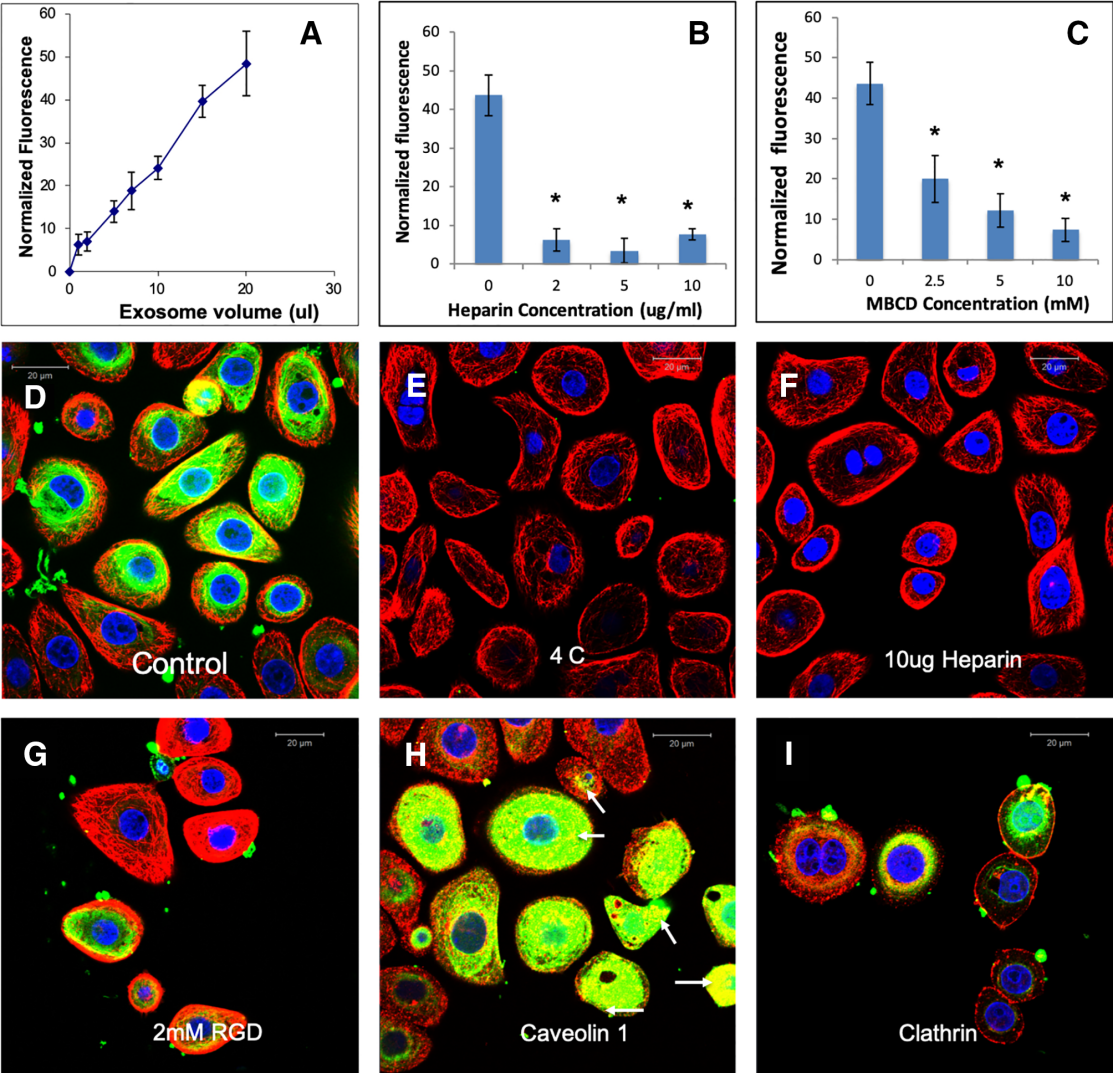
Endocytosis of human mesenchymal stem cell (HMSC) extracellular vesicles (EVs) by TIGK gingival keratinocytes: **(A)** Graphical representation of dose-dependent and saturable endocytosis of fluorescently labeled HMSC EV by TIGK cells. Data points represent mean fluorescence (n=6) +/− SD. The EV volume was standardized as described under the methods section. **(B)** Graph showing the dose dependent inhibition of HMSC EV endocytosis after pre-treatment of the EVs with heparin to block interaction with the cell surface HSPGs. Data represent mean percentage fluorescence with respect to control +/− SD. **(C)** Graph showing the reduction in HMSC endocytosis after disruption of target cell membrane cholesterol with varying doses of methyl-β-cyclodextrin (MBCD). Data is presented as mean percentage fluorescence with respect to control +/− SD. Representative confocal micrograph depicting the endocytosed fluorescently labeled HMSC EVs within target HMSCs after 1 h of incubation at 37°C. **(E)** Representative confocal micrograph indicating the abrogation of HMSC EV endocytosis when the experiment is performed at 4°C. **(F)** Representative confocal micrograph showing that pre-treatment of EVs with heparin blocks HMSC EV endocytosis. **(G)** Representative confocal micrograph of HMSC EV endocytosis after pre-treatment of the cells with 2 mM RGD peptide to block cell surface integrins. In images **(D**–**G)**, green fluorescence represents endocytosed EVs. Red fluorescence represents tubulin counter stain and blue fluorescence indicates 4′,6-diamidino-2-phenylindole (DAPI) nuclear stain. **(H)** Confocal micrograph showing colocalization of endocytosed HMSC EVs (green) with caveolin1 (red). **(I)** Confocal micrograph showing the absence of co-localization between endocytosed EVs (green) and clathrin (red). * represents statistical significance (P < 0.05, ANOVA followed by Tukey’s *ad-hoc*) with respect to control.

To test if HMSC EVs possess a common endocytic mechanism across different cell types, we evaluated the endocytosis of naïve HMSC derived EVs in two additional cell types namely: J774A.1 monocyte-like cells and TIGK gingival keratinocytes. Results presented in **Figures 4** and **5** indicate that HMSC EVs are endocytosed using a similar pathway and display dosage and temperature dependence. The involvement of HSPGs and the role of the caveolar pathway in the endocytic process was also common across the three cell types. The layout of images in **Figures 2**, **4**, and **5** have been maintained consistent for ease of comparison. Overall, these results indicate the existence of at least one common heparin-sensitive and caveolin-mediated mechanism of HMSC EV endocytosis across diverse cell types.

### Extracellular Vesicles From Differentiated Human Mesenchymal Stem Cells Induce Lineage Specific Differentiation of Naïve Human Mesenchymal Stem Cells *In Vitro* and *In Vivo*

Undifferentiated HMSCs in 3D cultures were incubated with EVs isolated from naïve and differentiated HMSCs for 72 h. Osteogenic, chondrogenic, and adipogenic EVs induced a significant increase in the expression levels of respective lineage specific marker genes with respect to untreated controls (**Table 1**). These genes included a mixture of growth factors, transcription factors, and ECM proteins representative of the individual lineages. No significant change in gene expression was observed with naïve HMSC EVs and the changes observed with lineage specific EVs were restricted to that specific lineage.

**Table 1:** Extracellular vesicle (EV) mediated lineage-specific differentiation of human mesenchymal stem cells (HMSCs) *in vitro*..

GENE	Cont. EV	Ost. EV	Cho. EV	Adi. EV
**Runx2**	1.11 +/- 0.09	3.80 +/- 0.06	0.99 +/- 0.06	0.79 +/- 0.02
**OSX**	1.27 +/- 0.09	2.91 +/- 0.05	1.64 +/- 0.17	0.81 +/- 0.05
**BMP2**	1.09 +/- 0.07	7.97 +/- 0.26	1.20 +/- 0.11	0.89 +/- 0.07
**BMP9**	1.52 +/- 0.07	10.29 +/- 3.61	1.45 +/- 0.12	1.39 +/- 0.06
**TGFb1**	0.99 +/- 0.04	0.98 +/- 0.03	4.90 +/- 0.02	0.83 +/- 0.07
**SOX9**	1.09 +/- 0.08	1.12 +/- 0.09	5.71 +/- 0.41	0.63 +/- 0.03
**COMP**	0.97 +/- 0.05	0.98 +/- 0.13	3.15 +/- 0.33	0.72 +/- 0.02
**COLL 2**	0.73 +/- 0.05	0.68 +/- 0.02	10.93 +/- 2.19	0.09 +/- 0.01
**PPARg**	0.79 +/- 0.10	1.14 +/- 0.03	0.62 +/- 0.09	4.27 +/- 0.19
**CEBPA**	1.20 +/- 0.29	1.37 +/- 0.14	1.81 +/- 0.11	6.81 +/- 0.84
**LPL**	0.94 +/- 0.07	1.21 +/- 0.22	1.11 +/- 0.21	4.34 +/- 0.27
**ADIPOQ**	1.10 +/- 0.07	1.27 +/- 0.10	1.61 +/- 0.17	2.47 +/- 0.19

Table represents fold changes in gene expression levels of representative marker genes for osteogenic, chondrogenic and adipogenic differentiation of HMSCs after treatment of naïve HMSCs for 72 h with the EVs isolated from naïve and respectively differentiated HMSCs. The data are presented as mean fold change +/− SD with respect to control (n = 4). Red lettering within the table denotes lineage specific genes.

**Figure 6:**
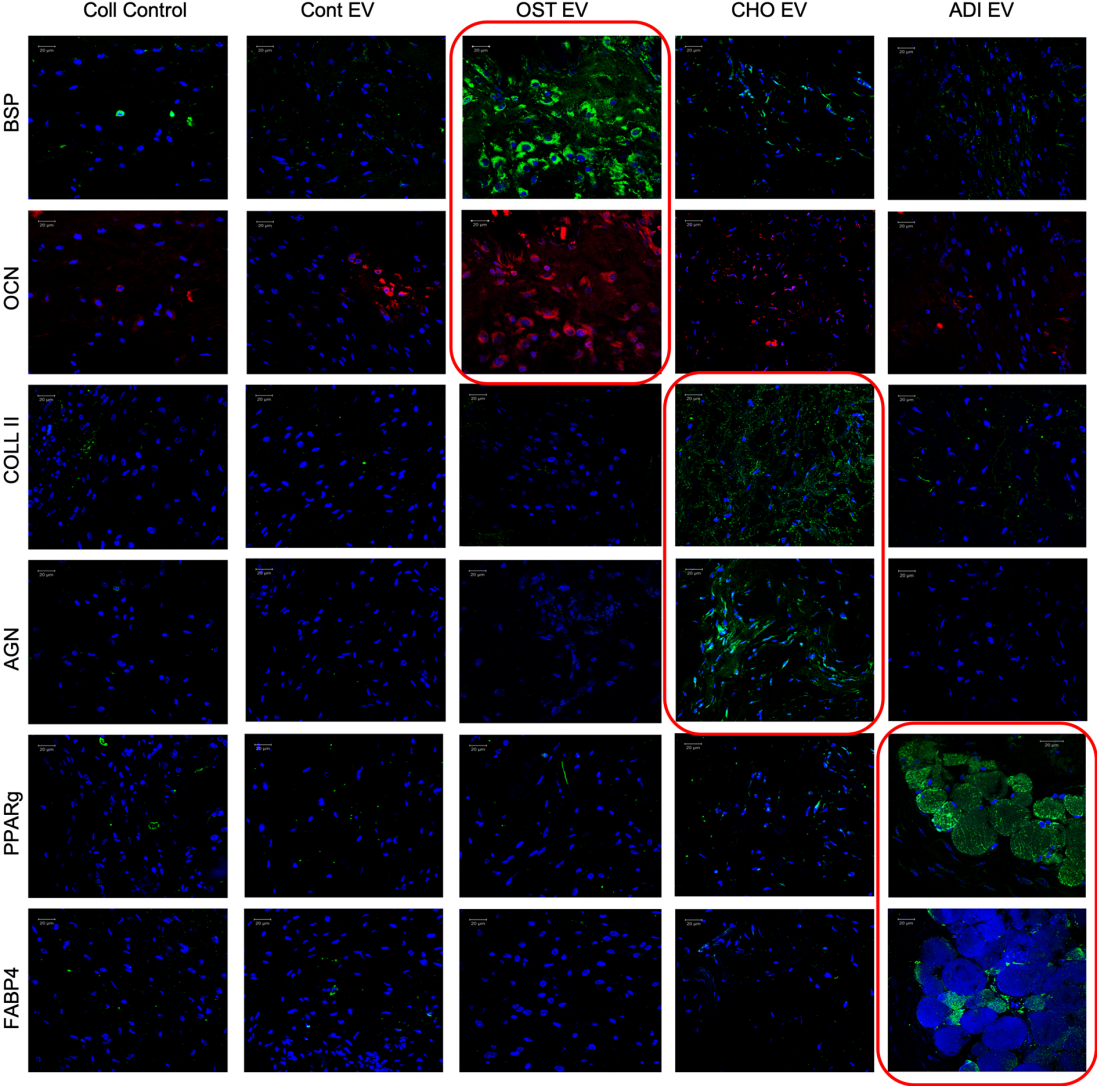
Lineage-specific differentiation of human mesenchymal stem cells (HMSCs) *in vivo:* Confocal micrographs representing immunohistochemical staining for the presence of lineage specific proteins in the tissue explants from the subcutaneous implantation of HMSCs with and without respective control and lineage specific EVs. The red boxed images represent lineage specific protein expression. In all images, blue represents DAPI nuclear staining and red or green represents the immunolabeled protein. Scale bar represents 20 µm in all images.

To verify these effects *in vivo*, collagen sponges loaded with undifferentiated HMSCs with or without EVs were implanted subcutaneously in the back of immunocompromised mice. After 4 weeks, the forming tissues were excised, fixed, embedded and the sections were analyzed by fluorescence immunohistochemistry for the expression of lineage-specific marker proteins. For all three different EVs, lineage-specific protein expression was observed. **Figure 6** shows representative confocal images of the sections. Similar to the qPCR results, the expression was lineage specific.

For osteogenic differentiation the expression levels of bone sialoprotein (BSP) and osteocalcin (OCN) were analyzed. Results presented in **Figure 6** show that HMSCs from the group treated with osteogenic EVs showed an increased presence of BSP and OCN compared to the control group as well as other EV treated groups adding evidence to the *in vitro* results presented in **Table 1**. Similarly, chondrogenic differentiation was evaluated by looking at the expression levels of type II collagen, a major component of the cartilaginous matrix as well as the expression level of aggrecan (AGN), a cartilage specific proteoglycan. Results presented in **Figure 6** show that type II collagen and AGN expression was elevated in HMSCs subjected to chondrogenic EV treatment with respect to the control group as well as the other EV treated groups. Finally, adipogenic differentiation of HMSCs from the subcutaneous implants was evaluated by the expression levels of peroxisome proliferator activator receptor-gamma (PPAR-γ) and fatty acid binding protein 4 (FABP4). PPAR-γ is a nuclear receptor that controls adipogenesis and adipogenic differentiation of HMSCs (Brun and Spiegelman, 1997; Spiegelman et al., 1997; Siersbaek et al., 2010). Results presented in **Figure 6** show an increased expression of PPAR-γ and FABP4 in HMSCs treated with adipogenic EVs compared to controls indicating an induction of adipogenic differentiation. Additionally, these cells demonstrate the presence of fat-like deposits arrows in **Figure 6**.

Collectively, these results indicate that EVs isolated from differentiating HMSCs can induce lineage-specific phenotypic changes in naïve HMSCs *in vitro* and *in vivo*. The verification of the *in vitro* results *in vivo* validated the lineage specificity of the EVs and additionally, also provided evidence that the effect that was observed after a few days *in vitro* translates into a long-term effect over a period of 4 weeks *in vivo*.

## Discussion

Regenerative strategies require the recruiting and instructing of cells to form new tissues. HMSC EVs are of current interest because they demonstrate immunomodulatory and regenerative potential that may rival the use of HMSCs or growth factors in regenerative medicine (Cheng et al., 2017). Furthermore, studies are currently underway to engineer HMSCs to improve their ability to produce EVs by altering several secretory pathways (Phan et al., 2018). The immunomodulatory, angiogenic, and regenerative potential of HMSC EVs is well documented (Lai et al., 2010; Reis et al., 2012; Mokarizadeh et al., 2012). Others and we have shown the potential of bone marrow derived HMSC EVs for regenerative medicine applications (Narayanan et al., 2016; Martins et al., 2016; Wang et al., 2018). Albeit the presence of multiple studies documenting the potential of MSC derived EVs, several aspects of their mechanisms that are translationally relevant and important remain as key knowledge gaps.

In this study, we have provided insights into some of the basic properties of HMSC derived EVs and how they may be utilized and exploited for improving tissue engineering strategies. We began by investigating HMSC EV endocytosis. Identification of the endocytic mechanism can provide valuable information to target EVs for therapeutic delivery. With respect to EV endocytosis, the clathrin pathway, caveolar pathway, phagocytosis, and even macropinocytosis have all been implicated in endocytosis of EVs (Mulcahy et al., 2014; Li et al., 2015; Alcayaga-Miranda et al., 2016). We observed energy dependence, dose dependence as well as dependence on membrane cholesterol indicating the involvement of the lipid raft/caveolar endocytic pathway. Furthermore, we show that the HMSC EVs are endocytosed in a manner that involves the target cell surface HSPGs. Based on our observations with dental pulp HMSC derived EVs, this appears to be a common endocytic mechanism for HMSC derived EVs (Huang et al., 2016). Further studies using different HMSC sources are required to conclusively determine if this mechanism is applicable to HMSCs in general.

In this study, we also show that the HMSC EVs are endocytosed by monocytes and keratinocytes using a similar endocytic process to that of recipient HMSCs *via* the cell surface HSPGs. We chose these two cell types as representative cells for hematopoietic and ectodermal cells respectively. Further studies with other representative cell types belonging to multiple germ layers is required to conclusively establish one common pathway across all cell types. However, this important first observation identifies a pathway that can be targeted to enhance the endocytic efficiency of therapeutic EVs. For example, the HIV TAT peptide and poly arginine repeats containing peptide sequences have been implicated to be endocytosed by target cells *via* HSPGs and in addition, HSPGs have been implicated as an accessible target receptor for delivery of biological cues to enable disease treatment as well as tissue regeneration (Fuchs and Raines, 2004; Fuchs and Raines, 2006; Christianson and Belting, 2014). These results and the results presented in this study indicate that it may be possible to enhance HMSC EVs endocytosis by target cells by tagging them with HSPG binding peptides. If possible, such modifications may promote enhanced delivery by reducing dosage as well as minimizing ectopic effects.

This study also explores an important question regarding the use of EVs for therapeutic purposes: Does the state of the parental cell influence EV functionality? The results presented here show that when HMSCs were differentiated into osteogenic, chondrogenic, and adipogenic lineages, the secreted EVs from these cells maintained their morphology and expression of exosomal surface markers. We next considered if lineage-specification of parental HMSCs would inform the differentiation potential of their respective EVs. Results indicated that the endocytic efficiency of HMSC EVs is not altered by changes to cell state. EVs isolated from osteogenic, chondrogenic, and adipogenic HMSCs did not show any significant difference in their dose-dependent ability to be endocytosed by naïve HMSCs. However, they were able to effect lineage specific changes within the target HMSCs *in vitro* and *in vivo*. We anticipate that this effect is due to the alterations to the exosomal cargo of miRNA, mRNA, and proteins. Our characterization of lineage specification by EVs from lineage differentiated HMSCs underscores the unique character of cell-type specific EVs. This novel finding that directing tissue-specific regeneration using EVs from differentiated HMSCs has wide-ranging applications in regenerative medicine.

Overall, the data presented in this study indicates that altering the HMSC cell state generates EVs with function-specific properties without altering EV characteristics, size distribution, or endocytic ability. Furthermore, HMSC EVs were endocytosed by cell types from other germ layers in a similar dose-dependent and pathway-specific manner identifying a common mechanism of endocytosis. These results identify underlying mechanism and properties of HMSC derived EVs and provide an indication to how they may be manipulated for various applications in disease treatment and regenerative medicine.

## Data Availability Statement

The datasets generated for this study are available on request to the corresponding authors.

## Ethics Statement

The animal study was reviewed and approved by the UIC Animal Care Committee (ACC).

## Author Contributions

C-CH is the first author and designed and performed most of the experiments. MK performed the experiments with the monocytes in LC’s laboratory. RN was the technician that assisted C-CH in his experiments. LD’s laboratory provided the keratinocytes and performed the endocytosis experiments using them. PG performed the *in vivo* experiments and edited the manuscript. SR conceptualized the original hypothesis, directed C-CH and RN, performed the imaging analyses, and wrote the manuscript.

## Funding

This work was funded by NIH R01DE027404 and the Osteology foundation advanced researcher award to SR and PG (Award # 17-106) and NIH R01GM50875 to LD.

## Conflict of Interest

The authors declare that the research was conducted in the absence of any commercial or financial relationships that could be construed as a potential conflict of interest.

## Supplementary Material

Supplementary Figure 1 Cell viability: **(A)** Representative fluorescent images from live/dead cell assay (Life Technologies) performed on HMSCs cultured in the presence of growth medium (GM) and serum-free medium (SFM) for 24 hours. **(B)** represents results from MTS assay (Promega) performed on HMSCs cultured in the presence of GM or SFM (n=6) for 24 hours. Data in B represent mean +/- SD. No statistically significant difference was found between the two groups as measured by student’s t-test.
