# Supplementary figures and images for: Evaluating the Endocytosis and Lineage-Specification Properties of Mesenchymal Stem Cell Derived Extracellular Vesicles for Targeted Therapeutic Applications
Source: Front Pharmacol. 2020 Mar 3;11:163. doi: 10.3389/fphar.2020.00163 (PMC7063066; doi:10.3389/fphar.2020.00163)

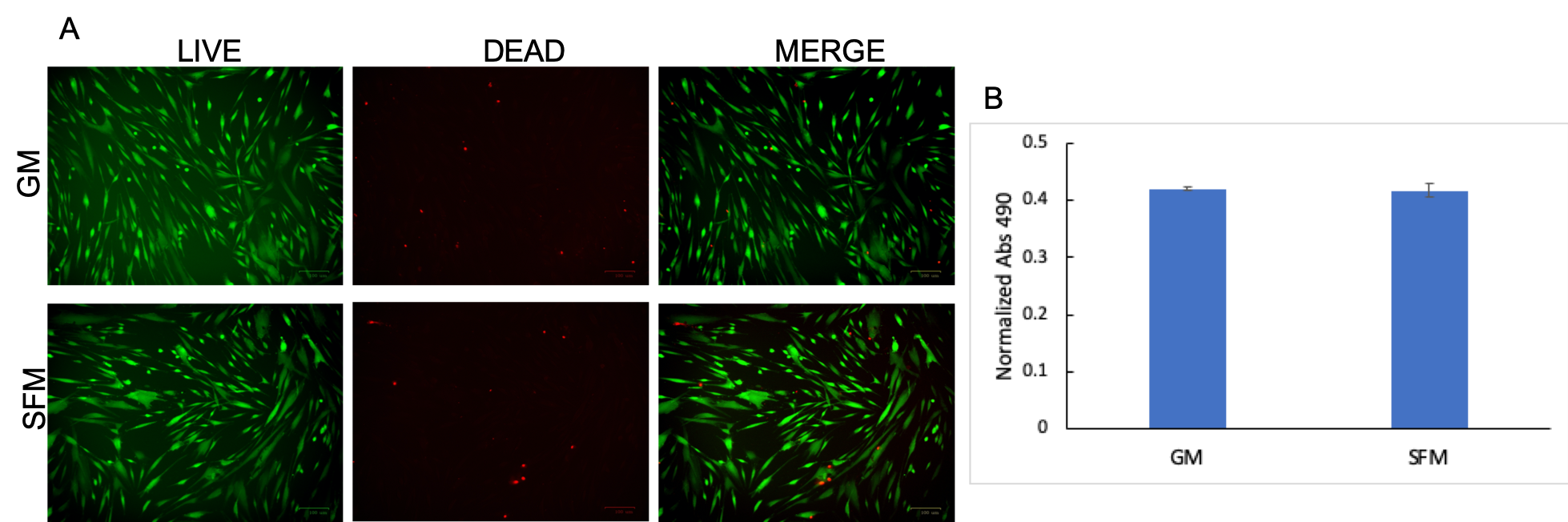

Supplement: Supplementary Figure 1 — Cell viability: (A) Representative fluorescent images from live/dead cell assay (Life Technologies) performed on HMSCs cultured in the presence of growth medium (GM) and serum-free medium (SFM) for 24 hours. (B) represents results from MTS assay (Promega) performed on HMSCs cultured in the presence of GM or SFM (n=6) for 24 hours. Data in B represent mean +/- SD. No statistically significant difference was found between the two groups as measured by student’s t-test. [file Image_1.png]
